# Predicting Visual Outcomes for Macula-Off Rhegmatogenous Retinal Detachment with Optical Coherence Tomography

**DOI:** 10.1155/2014/269837

**Published:** 2014-12-11

**Authors:** Noriyuki Suzuki, Hiroshi Kunikata, Naoko Aizawa, Toshiaki Abe, Toru Nakazawa

**Affiliations:** ^1^Department of Ophthalmology, Tohoku University Graduate School of Medicine, Sendai 980-8574, Japan; ^2^Department of Retinal Disease Control, Tohoku University Graduate School of Medicine, Sendai 980-8574, Japan; ^3^Division of Clinical Cell Therapy, Tohoku University Graduate School of Medicine, Sendai 980-8575, Japan; ^4^Department of Advanced Ophthalmic Medicine, Tohoku University Graduate School of Medicine, Sendai 980-8574, Japan

## Abstract

*Purpose.* We evaluated the ability of novel optical coherence tomography (OCT) parameters to predict postoperative best-corrected visual acuity (BCVA) in macula-off rhegmatogenous retinal detachment (RRD) eyes. *Methods.* We reviewed the medical records of 56 consecutive eyes with macula-off RRD. Clinical findings were analyzed including the relationship between preoperative OCT findings and 6-month postoperative BCVA. *Results.* Six-month postoperative BCVA was significantly correlated with preoperative findings including retinal height at the fovea, total and inner layer cross-sectional macular area within 2 mm of the fovea, and preoperative BCVA (*P* < 0.001, *P* < 0.001, *P* = 0.001, and *P* < 0.001, resp.). Multiple regression analysis revealed that the duration of macular detachment and total cross-sectional macular area were independent factors predicting 6-month postoperative BCVA (*P* = 0.024 and *P* = 0.041, resp.). *Conclusions.* Measuring preoperative total cross-sectional area of the macular layer within 2 mm of the fovea with OCT is a useful and objective way to predict postoperative visual outcome in eyes with macula-off RRD.

## 1. Introduction

Although remarkable progress has been achieved in the surgical treatment of eyes with rhegmatogenous retinal detachment (RRD), it is still difficult to predict postoperative visual outcomes when RRD includes a detached macula, known as macula-off RRD. Newly developed less-invasive surgical interventions, particularly 25-gauge microincision vitrectomy surgery (25GMIVS), have led to a very high initial reattachment rate for eyes with RRD, currently about 95% [1–5]. However, in eyes with macula-off RRD, degeneration of the photoreceptors in the detached area of the macula often prevents complete recovery of visual function and leads to central visual dysfunction, even after successful reattachment [5, 6].

Photoreceptor apoptosis has been reported to mainly occur within 3 days of RRD onset in experimental animal models [7, 8] and to induce the expression of various cytokines and chemokines [9, 10]. Clinically, many cases of macula-off RRD undergo postoperative atrophy of the outer macular layer after reattachment. Accordingly, a few reports have performed qualitative analysis of changes in the structure of the detached macular area represented in optical coherence tomography (OCT) images and have examined the potential role of such analysis in determining the postoperative visual prognosis of eyes with macula-off RRD [11, 12]. However, to the best of our knowledge, there are no current reports investigating the potential of quantitative OCT measurement parameters of the macula to serve as prognostic indicators of visual outcome.

We hypothesized that preoperative macular volume reflected photoreceptor apoptosis and could thus predict postoperative outcomes in eyes with macula-off RRD. In order to test this hypothesis, we developed new OCT parameters that could provide a suitable evaluation of the macular structure and then determined the relationship between these parameters and postoperative visual outcomes in macula-off RRD eyes.

## 2. Patient and Methods

### 2.1. Subjects

We performed a retrospective analysis of the medical records of 56 consecutive eyes with macula-off RRD that underwent surgical intervention with a 25-gauge trocar cannula system or scleral buckling from January 2011 to February 2014 at Tohoku University Hospital. Eyes were included only if complete reattachment of the RRD was achieved after initial surgery. Eyes were excluded if they had prior vitreoretinal surgery, proliferative retinopathy, retinal vascular disease, chorioretinal atrophy, or high myopia (more than −10 diopters), if we could not obtain clear preoperative OCT measurements due to a bullous RRD or vitreous opacity, or if a single OCT scan could not capture the detached fovea and the retinal pigment epithelium layer. After the purpose and procedures of the operation were explained, informed consent was obtained from all patients. The procedures conformed to the tenets of the Declaration of Helsinki, and the study was approved by the Institutional Review Board of Tohoku University Graduate School of Medicine.

### 2.2. Measurements of Clinical Findings

**Figure 1:**
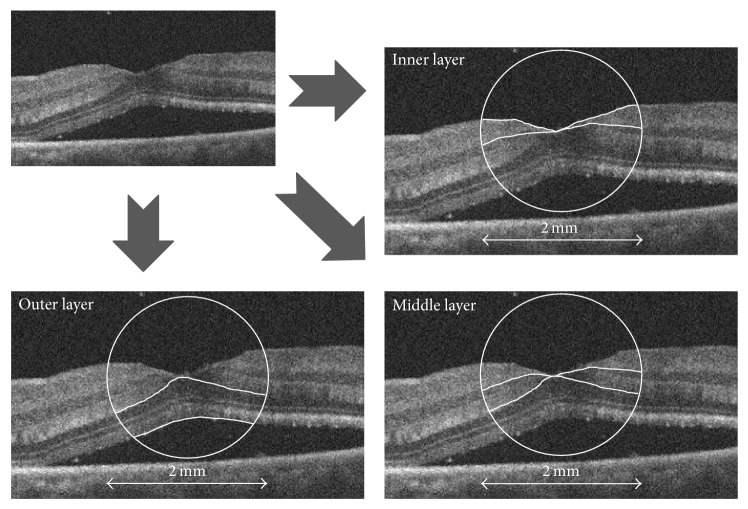
Preoperative optical coherence tomography (OCT) images. A circle with a diameter of 2 mm was manually centered at the foveal surface center of the detached macula in the OCT image. The macular area within the circle was divided into three sections: the inner layer (upper right: nerve fiber layer and ganglion cell layer), middle layer (lower right: inner plexiform layer and inner nuclear layer), and outer layer (lower left: outer plexiform layer and outer nuclear layer).

All patients underwent a complete ocular examination 6 months after surgery. Best-corrected visual acuity (BCVA) was measured preoperatively and 1 and 6 months postoperatively with the Landolt C visual acuity chart. Decimal acuity values were converted to logarithm of the minimal angle of resolution (log MAR) units. The detached macula was examined with spectral-domain (SD) OCT (Cirrus OCT, Carl Zeiss Meditec) in all patients preoperatively, and foveal thickness was also measured 1 and 6 months postoperatively. To evaluate the detached macula preoperatively, a 2 mm circle was manually centered on the surface of the fovea in a cross-sectional OCT macular image. The image of the macula within this circle was then manually segmented into three layers: the inner layer (nerve fiber layer and ganglion cell layer), middle layer (inner plexiform layer and inner nuclear layer), and outer layer (outer plexiform layer and outer nuclear layer) (Figure 1). All OCT images were horizontal scans. Postoperative foveal thickness was defined as the value in the central 1000 *μ*m area and was automatically calculated by the onboard OCT software. Our analysis of preoperative OCT parameters included retinal detachment (RD) height (the vertical distance from the detached fovea to the retinal base) [12] and macular cross-sectional area within a 2 mm circle centered on the foveal surface center in the OCT image. Separate values were also recorded for cross-sectional area in three macular layers.

### 2.3. Statistical Analyses

All statistical analysis was performed with JMP software (Pro version 10.0.2, SAS Institute Japan Inc., Tokyo, Japan). The correlation of 6-month postoperative BCVA to preoperative characteristics and operative, visual, and anatomical outcomes was determined with Spearman's rank correlation coefficient. Significant differences between preoperative and 1- and 6-month postoperative BCVA were determined with the paired *t*-test. Independent variables affecting 6-month postoperative BCVA were determined with multiple linear regression analysis. The significance level was set at *P* < 0.05.

## 3. Results

**Figure 2:**
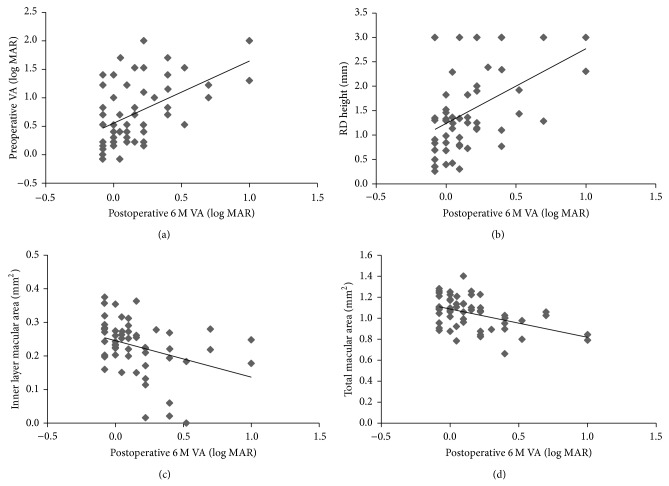
Correlation of preoperative clinical findings to 6-month postoperative best-corrected visual acuity (BCVA). There was a positive correlation between preoperative and 6-month postoperative BCVA ((a); *r* = 0.48, *P* < 0.001). There was also a positive correlation between retinal detachment height and 6-month postoperative BCVA ((b); *r* = 0.47, *P* < 0.001). There was a negative correlation between the cross-sectional area of the inner macular layer and 6-month postoperative BCVA ((c); *r* = −0.43, *P* = 0.001). There was also a negative correlation between total macular cross-sectional area and 6-month postoperative BCVA ((d); *r* = −0.44, *P* < 0.001).

Table 1 shows the possible association of preoperative characteristics and operative, visual, and anatomical outcomes with 6-month postoperative BCVA in 56 eyes with RRD. Lens-sparing 25-gauge vitrectomy was performed in patients younger than 50 years. Cataract progression, which could have affected visual acuity, did not occur in any of these patients 6 months after surgery. Six-month postoperative BCVA was positively correlated with preoperative BCVA, 1-month postoperative BCVA, and preoperative RD height (*P* < 0.001, *P* < 0.001, and *P* < 0.001, resp.; Table 1 and Figure 2). Six-month postoperative BCVA was negatively correlated with 1-month postoperative foveal thickness and preoperative total and inner layer cross-sectional macular area (*P* = 0.006, *P* < 0.001, and *P* = 0.001, resp.; Table 1 and Figure 2). Six-month postoperative BCVA was significantly higher than preoperative BCVA and 1-month postoperative BCVA (*P* < 0.001 and *P* < 0.001, resp.). Multiple regression analysis revealed that the duration of the macular detachment and the total cross-sectional macular area were independent factors predicting 6-month postoperative BCVA (*P* = 0.024 and *P* = 0.041, resp.; Table 2).

**Figure 3:**
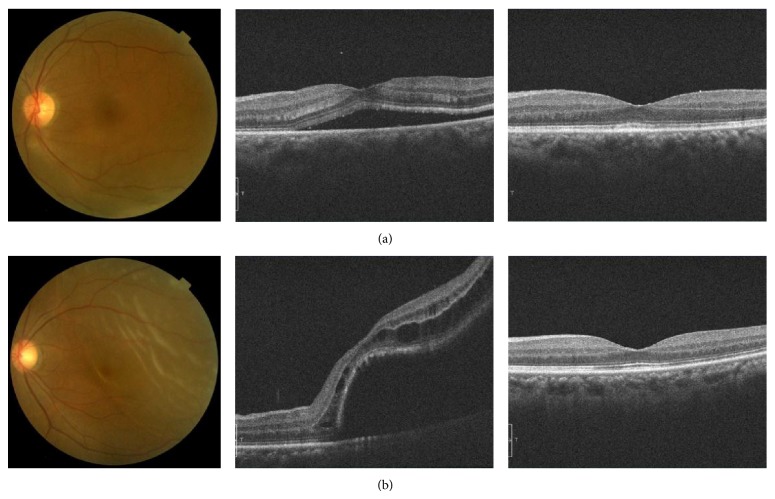
Representative eyes with good and poor visual outcomes after surgery for macula-off rhegmatogenous retinal detachment. (a) 65-year-old woman (preoperative decimal visual acuity: 0.7) with a good visual outcome (postoperative decimal visual acuity: 1.2). (b) 60-year-old woman (preoperative decimal visual acuity: 0.3) with a poor visual outcome (postoperative decimal visual acuity: 0.3). Preoperative photographs of the fundus, preoperative optical coherence tomography (OCT) images, and postoperative OCT images are shown on the left, center, and right, respectively. Preoperative foveal area was relatively thinner in the case with a poor outcome than in the case with a good outcome.

Images of eyes representing good and poor visual outcomes after surgery for macula-off RRD are shown in Figure 3.

## 4. Discussion

We set out to evaluate the potential of newly developed OCT parameters to predict postoperative BCVA in macula-off RRD eyes. We found that 6-month postoperative BCVA was significantly correlated with RD height at the fovea and with the total and inner cross-sectional area of the macular layer within 2 mm of the fovea, as well as with preoperative BCVA. Furthermore, multiple regression analysis revealed that the duration of the macular detachment and the total cross-sectional macular area were independent factors predicting 6-month postoperative BCVA.

Surgeons cannot easily predict postoperative visual outcomes in cases of RRD, and even after successful RRD surgery, many patients only regain a poor level of postoperative visual function. This often causes patients to experience preoperative anxiety. Our results confirmed existing data showing that the duration of the macular detachment was associated with postoperative visual outcome, although the usefulness of this parameter is limited, because it depends on the memory of the patient and their cooperation and therefore cannot always be reliably known [13–15]. Though there are many existing reports showing that early postoperative OCT macular findings are associated with final visual function [16–25], there are only a few reports examining preoperative structural changes in the macula using up-to-date SD-OCT imaging of the detachment and the association of these changes with visual outcomes [11, 12]. These studies found that qualitatively measured preoperative characteristics of RRD eyes, such as intraretinal separation and outer layer undulation, were associated with a higher postoperative incidence of disruption to the photoreceptor inner/outer segment junction and the presence of external limiting membranes, causes of poor visual outcomes. Qualitative measurements are, however, subjective and prone to error, creating the need for quantitative methods to measure the detached macula with OCT, in order to find prognostic indicators of final visual outcome in RRD eyes.

The pathogenesis of poor visual outcomes in RRD is related to photoreceptor cell death [7–10], suggesting that the number of surviving retinal cells in the fovea should be the ideal prognostic indicator of final visual outcome. The three-dimensional (3D) volume of the macula, which is measureable by recent advanced OCT techniques, is closely associated with the number of surviving cells but would only be feasible to measure in young patients with flat RRD. In patients with bullous RRD, the detached macula is unstable and shifts its position in the vitreous faster than the scanning speed of existing 3D OCT devices, making it impossible to evaluate foveal volume in these eyes. To overcome this technical difficulty, we used two-dimensional OCT to measure the cross-sectional area of the macular layer in a 2 mm circle centered on the fovea and investigated its potential as an indicator of final BCVA. We developed this measurement parameter after observing that the detached section of the macula is not straight or flat in OCT images but instead lies obliquely across the image plane. Conventionally measuring cross-sectional area in a square or rectangle horizontal to the choroid would thus tend to overestimate foveal thickness. By contrast, measurements of cross-sectional area in a 2 mm circle centered on the fovea (which is about 1 mm in diameter) should be reliable regardless of the orientation or position of the detachment. A circular area larger than 2 mm would begin to lose reliability, as it would be more influenced by intraretinal edema, bending, or severe undulation of the detachment. Thus, we believe that it is most reasonable to adopt the cross-sectional area of the macular layer within 2 mm of the fovea as an indicator of macular health.

This study showed that RD height at the fovea, a measurement parameter used in a number of earlier studies, was associated with postoperative BCVA in a single regression analysis, confirming earlier reports [11, 12]. This is an understandable result, as when the distance between the retinal pigment epithelium and the photoreceptors increases, the foveal cones receive less oxygenation and nutrition from the choroid and photoreceptor cell degeneration increases. However, multiple regression analysis revealed that it was not an independent factor predicting 6-month postoperative BCVA (*P* = 0.203). The cause of this discrepancy is unclear but may have been related to the instability of RD height, particularly in bullous RRD and particularly in older eyes, because the detached macula can more easily shift its position in the vitreous. It is difficult to accurately and reproducibly evaluate RD height in such eyes, leading us to speculate that preoperative RD height cannot be considered a reliable predictor of postoperative visual function in eyes with macula-off RRD.

Limitations of this study included a relatively short follow-up time of 6 months, a relatively small sample size (about 60), and the omission of postoperative functional findings from standard automated perimetry or focal electroretinography. Additionally, although bullous RRD eyes are often seen in the clinic, the method described here cannot be used to predict postoperative outcomes in cases when OCT scans do not show the macula. Furthermore, to prevent bias in the results, it was necessary to omit the inner macular layer in the cross-sectional image from our multiple regression analysis, because the total and inner layer values were not independent, both being OCT findings and being closely correlated with 6-month postoperative BCVA. At first, we hypothesized that visual outcome would be associated with the area of the outer macular layer, as this contains the outer nuclear layer and the photoreceptor cells, but this hypothesis was not borne out by the data. It is unclear why this was so, but it may have been related to the susceptibility of the outer layer to intraretinal edema or undulation, which makes it difficult to obtain accurate measurements. Nevertheless, we believe our results show that simple OCT measurement of total cross-sectional area within 2 mm of the fovea is currently the most useful and objective way to predict postoperative visual outcomes in eyes with macula-off RRD, at least until technology to quickly evaluate macular volume in three dimensions becomes available for use in eyes with detached maculas. The usefulness of the measurement method described here would also be greatly enhanced by an OCT program to automatically measure cross-sectional macular area in eyes with macula-off RRD.

In conclusion, OCT measurement of preoperative total cross-sectional area of the macular layer within 2 mm of the fovea is a useful and objective way to predict postoperative visual outcomes in eyes with macula-off RRD and was closely correlated with 6-month postoperative BCVA. Further investigation is needed to measure the macular volume and determine its relationship with visual outcomes, which could lead to the development of a new automatic OCT program.

## Figures and Tables

**Table 1:** Preoperative characteristics and operative, visual, and anatomical outcomes of 56 eyes with rhegmatogenous retinal detachment and their possible association with 6-month postoperative visual acuity.

		*r*	*P* value
Number of eyes	56	—	—
Age (years)	50.0 ± 19.8	0.09	0.524^a^
Sex (*n*, %)		—	0.557^b^
Male	38, 67.9%	—	—
Female	18, 32.1%	—	—
Spherical equivalent (diopter)	−3.15 ± 2.67	−0.01	0.950^a^
Duration of macular detachment (days)	33.3 ± 72.7	−0.09	0.565^a^
Procedure (*n*, %)		—	0.935^b^
PPV only	10, 17.7%	—	—
PPV with cataract surgery	27, 48.2%	—	—
Scleral buckling	19, 33.9%	—	—
Visual course (decimal VA)			
Preoperative	0.19 ± 0.27	0.48	<0.001^a^
1 M postoperative	0.53 ± 0.51	0.82	<0.001^a^
6 M postoperative	0.69 ± 0.55	—	—
Pre-op OCT findings			
RD height (mm)	1.45 ± 0.87	0.47	<0.001^a^
Total macular area (mm^2^)	1.05 ± 0.16	−0.44	<0.001^a^
Outer layer macular area (mm^2^)	0.62 ± 0.13	−0.17	0.221^a^
Middle layer macular area (mm^2^)	0.19 ± 0.06	−0.04	0.759^a^
Inner layer macular area (mm^2^)	0.23 ± 0.08	−0.43	0.001^a^
Post-op OCT findings			
1 M postoperative FT (*μ*m)	269.9 ± 79.7	−0.37	0.006^a^
6 M postoperative FT (*μ*m)	255.4 ± 35.7	−0.24	0.096^a^

FT = foveal thickness, OCT = optical coherence tomography, PPV = pars plana vitrectomy, RD = retinal detachment, and VA = visual acuity.

^a^Spearman's correlation coefficient by rank test, ^b^unpaired *t*-test.

**Table 2:** Multiple regression analysis for independent factors contributing to 6 M postoperative VA.

Variable			*β*	*P* value
Dependent	Independent		
Postoperative VA	Age		0.041	0.784
	Duration of macular detachment		0.869	0.024
	Preoperative VA		0.188	0.249
	Preoperative OCT findings	RD height	0.212	0.203
		Total macular area	−0.511	0.041
		Outer layer macular area	0.334	0.180
		Middle layer macular area	0.267	0.156

VA = visual acuity, OCT = optical coherent tomography, RD = retinal detachment, and *β* = standard partial regression coefficient.
